# Variation in Exon 29 of the NOS1 Gene Does Not Contribute to Parkinson’s Disease in the North Karnataka Population

**DOI:** 10.7759/cureus.45347

**Published:** 2023-09-16

**Authors:** Rudragouda Bulagouda, Smita Hegde, Rajat Hegde, Ashwini Hiremath, G M Wali, Gurushantappa S Kadakol

**Affiliations:** 1 Anatomy, BLDE (Deemed to be University) Shri B M Patil Medical College, Hospital and Research Centre, Vijayapura, IND; 2 Genetics, Karnataka Institute for DNA Research, Dharwad, IND; 3 Neurology, BLDE (Deemed to be University) Shri B M Patil Medical College, Hospital and Research Centre, Vijayapura, IND; 4 Neurology, Neurospecialist Centre, Belagavi, IND; 5 Human Genetics Laboratory, Department of Anatomy, BLDE (Deemed to be University) Shri B M Patil Medical College, Hospital and Research Centre, Vijayapura, IND

**Keywords:** nos1, 3’utr, molecular analysis, north karnataka, parkinson's disease

## Abstract

Introduction: Nitric oxide (NO) overproduction has been found to have neurotoxic effects on the brain. Moreover, in 1-methyl-4-phenyl-1,2,3,6-tetrahydropyridine (MPTP) induced, the suppression of the NO-synthesizing enzymes, such as neuronal nitric oxide synthase (nNOS) and inducible NOS (iNOS), has neuroprotective benefits in Parkinson's disease (PD). These findings imply that NOS may have a role in regulating the nigral dopaminergic neurons' tolerance to environmental stressors in PD.

Objective: In the present study, we investigated variations in the *NOS1* gene that may raise the likelihood of PD.

Methods: PD patients who visited the neurology departments of several medical colleges and hospitals in North Karnataka, India, between 2009 and 2011 were included in the study. The detailed clinic pathological details were obtained from 100 PD patients. Genomic DNA was isolated using the kit method followed by the evaluation of the quality and quantity of isolated gDNA. Polymerase chain reaction (PCR) amplification of exon 29 was performed, and sequencing was performed using the Applied Biosystems* *ABI 3500 Sanger sequencing platform.

Results: The present study is comprised of 100 PD patients, which includes 65 males and 35 females. There were 64 sporadic, 34 idiopathic, and two familial PD cases. The majority (67.1%) of PD cases were from metropolitan areas. Community-based segregation showed that the maximum cases were from Hindu Lingayat. A proportion (90.8%) of the patients had tremors, 32.7% of them displayed slowness in their daily tasks, and 8.1% of them had dyskinesia. Molecular analysis showed two untranslated region (UTR) variations g.151787 del T (rs1434015950) and g.151745 C>T (rs2682826) in our study group.

Conclusion: The absence of mutations in the targeted *NOS1* gene in the PD patients from North Karnataka shows the involvement of other genes in the molecular pathophysiology. Thus, it is crucial to screen other possible genes using cutting-edge technology to obtain a clear picture of the genetics of PD.

## Introduction

Parkinson's disease (PD) is a neurological condition that progresses and manifests as tremors, bradykinesia, rigidity, and postural instability. A wide range of non-motor symptoms is present in PD. In the substantia nigra area of the brain, dopaminergic neurons primarily experience progressive degradation in PD [1,2]. The substantia nigra is responsible for directing motion. It provides dopamine-mediated excitatory and inhibitory massages to the putamen and caudate nucleus, which control regulatory movements [3]. 

The development of alpha-synuclein insoluble proteins leads to the formation of Lewy bodies, which are a major pathogenic feature of PD. PD, which is chronic and progressive, is also known as a movement disorder. The main symptoms are brought on by excessive muscle contraction, which is typically a result of insufficient levels of dopamine, produced by the brain's dopaminergic neurons. High-level cognitive impairment and subtle linguistic issues are examples of secondary symptoms. In certain instances, the symptoms are brought on by toxicity, medications, genetic mutations, brain injuries, or other medical conditions [4,5]. 

PD has a wide range of etiologies. A proportion (90%) of the instances are believed to be sporadic, and 10% have genetic roots. PD inherits its genetic makeup in a Mendelian manner. Patients with family variants of PD have been shown to have several genetic alterations [6]. Clinically, it might be difficult to distinguish between familial and idiopathic PD. However, patients with familial illness exhibit specific characteristics that are not present in those with idiopathic PD, such as the young age at which the disease first manifests itself (between 30 and 60 years) [7,8]. The precise origin and pathogenesis of PD are yet unknown. Current research indicates that PD may be caused by certain mutations [9]. Genes, such as *NOS1* (nitric oxide synthase 1), *SNCA* (alpha-synuclein), *PINK1 *(PTEN-induced putative kinase 1),* Parkin*, *UCH-L1 *(ubiquitin C-terminal hydrolase L1), *DJ-1 *(PARK7), *NR4A2* (nuclear receptor subfamily 4, group A, member 2), and *LRRK2* (leucine-rich repeat kinase 2) have all been found to be mutated in PD [10,11,12,13], although only 1% of PD cases have *SNCA* mutations [14]. Previous research conducted in 2011-2012 on North Karnataka PD patients showed no genetic alterations in the *SNCA* gene [15]. The present study was conducted to evaluate the molecular changes in exon 29 of the *NOS1* gene in North Karnataka.

## Materials and methods

A total of 100 PD patients from both urban and rural areas who visited the neurology departments of medical colleges and hospitals in North Karnataka were enrolled in this study. PD patients with other comorbidities and those who did not agree to give samples were excluded from the study. Detailed clinical and demographic data were noted from each patient. To quantify the physical activity, tremor, stiffness, bradykinesia, and akinesia, acknowledged standard definitions were applied [16].

Clinical sample (blood) collection

The individuals who were enrolled in the study provided their written consent. Peripheral blood samples (1 mL) were obtained after taking informed consent from the patients and kept at 4°C in ethylenediaminetetraacetic acid (EDTA)-coated vacutainers. Blood samples from controls with similar racial backgrounds were also taken in addition to those from the patient.

DNA isolation

Genomic DNA was extracted from 300 μL of peripheral blood using a DNeasy blood and tissue kit (QIAGEN, Germany) as per manufacturer guidelines, and isolated genomic DNA was quantified using the Quawell Q3000UV Spectrophotometer (NanoDrop, Thermo Fisher Scientific Waltham, Massachusetts, United States). Quality was assessed using 0.8% agarose gel electrophoresis.

Mutation analysis

The cycle conditions used for the amplification were as follows: initial denaturation at 95°C for one minute, followed by 35 cycles of denaturation at 30°C for 0 seconds, annealing at 60.4°C for one minute, extensions at 68°C for one minute, and final extension at 68°C for five minutes. The *NOS1* gene (NG 011992.1) was amplified using designed primers (Table 1). The master mix composition used for the amplification was 7.35 μL of molecular-biology-grade water, 1.0 μL of 1x Taq buffer, 100 mM 0.2 μL of deoxynucleotide triphosphates (dNTPs), 10 pM 0.2 μL of forward primer, 10 pM 0.2 μL of reverse primer, 1.0 μL template DNA, and 1 U 0.05 μL of Taq polymerase, which were used to amplify a targeted region of interest. The amplified PCR product was seen using 1.5% agarose gel electrophoresis. Using an Applied Biosystems ABI 3500 Sanger sequencing platform (ABI 850 Lincoln Centre Drive, Foster City, Thermo Fisher Scientific) and BigDye Terminator v3.1 Cycle Sequencing Kit (Applied Biosystem, USA), purified PCR products of the *NOS1* gene were sequenced. Sequencing data were analyzed using Sanger sequence analysis software V5 (Applied Biosystem, USA).

**Table 1:** Primer sequence details used for the NOS1 (exon 29) gene amplification.

Forward primer	Reverse primer	Product size	Tm
TGCTGCGATGCAATGCTTTT	GACTTCAGTGGCTGAGGGAC	208	60.4 °C

## Results

This study is comprised of 100 participants with PD who had received a clinical diagnosis; 65 were males and 35 were females. There were 64 sporadic, 34 idiopathic, and two familial PD cases among the 100 individuals. Only 32.9% of the participants came from rural areas, while the majority (67.1%) were from metropolitan areas. According to the subjects' community-based segregation, the Hindu Lingayat made up 47.6% of the sample, followed by Brahmin (15.1%) and Muslims (10.6%) (Table 2).

**Table 2:** Baseline characteristics of Parkinson's disease patients in the study population

Clinico-demographical characteristics		n (%)
Gender	Male	65 (64.8)
	Female	35 (35.2)
Nature of Parkinson's disease	Sporadic	64 (64.5)
	Idiopathic	34 (34.3)
	Familial	2 (1.3)
Regional distribution	Urban	67 (67.1)
	Rural	32 (32.9)
Cast-wise distribution of Parkinson's disease	Lingayat	47 (47.6)
	Brahmin	15 (15.1)
	Muslim	10 (10.6)
	Others	25 (26.8)

Most of the PD patients were between the ages of 60 and 79 (54.2%), followed by those between 40 and 59 (37.2%). Individuals between the ages of 80 and 92 and between 20 and 39 were considerably less (Table 3).

**Table 3:** Age-wise distribution of the Parkinson's disease patients in the study population

Age (years)	N (%)
20 to 39	3 (2.9)
40 to 59	37 (37.2)
60 to 79	55 (54.2)
80 to 92	5 (5.7)

Table 4 lists the clinical signs of PD in the study participants. The percentage of patients who had tremors was around 90.8%, while 32.7% of them displayed slowness in their daily tasks and 8.1% of them had dyskinesia. Less than 5% of the patients had additional characteristics, which included trouble walking, bradykinesia, stiffness, weakness, frequent falls, and difficulty speaking (Table 4).

**Table 4:** Clinical features of Parkinson's disease; n=100 (%)

Parameter	n (%)
Tremors	90 (90.8)
Slowness in activities	32 (32.7)
Dyskinesia	8 (8.1)
Walking difficulty	5 (5.2)
Bradykinesia	3 (3.6)
Rigidity	3 (3.4)
Weakness	3 (2.7)
Frequent falls	1 (0.5)
Difficulty in speech	1 (0.2)

All the electropherograms of the PD samples were analyzed and evaluated with the control samples. The 29th exons of the *NOS1* gene were compared to the control samples. In the present study, only the g.151787 del T (rs1434015950) and g.151745 C>T (rs2682826) variants in the 3’UTR region were recorded. No coding sequence variants were recorded in our study (Table 5).

**Table 5:** Mutation recorded in exon 29 of the NOS1 gene in our study of Parkinson's disease (PD) patients.

GDNA Ng_011991.2	Mutation details	Variant type
g.151787 delT	rs1434015950SNP	3’ UTR
g.151745 C>T	rs2682826SNP	3’ UTR

## Discussion

Many efforts have been undertaken to comprehend the genetic causes of neurodegeneration. According to reports, PD affects more than 1.5% of people over the age of 60 and around 5% of people over the age of 80. Based on this, it is estimated that 0.32 million people in India have PD, compared to 1.2 million people in the Western population [15]. However, certain research performed in India revealed that the incidence of PD in the Indian population is lower compared to that in the Western population. The alarming rise in the prevalence of PD in India has been related to the demographic trend, changing environment, and lifestyle. According to recent investigations, there were 134 cases of PD among those over 50 [15]. The inhabitants of South India, and more especially the populations of North Karnataka, are a singular admixture of individuals from various ethnic, genetic, tribal, cultural, religious, and socioeconomic backgrounds. The maintenance of gene pools across the various populations of South India is a result of the caste endogamy and clan exogamy that are being practiced today. The practice of consanguinity (inbreeding), caste endogamy, and the conserved gene pool may have all contributed to the accumulation of mutations in functioning genes that raised the prevalence of genetic illnesses.

Recently, scientists at Duke University and the University of Miami demonstrated that two *NOS *gene variations are risk factors for PD. Moreover, they have demonstrated that several of these gene variations can interact with known environmental risk factors for PD (smoking and pesticide exposure), which may affect PD susceptibility [16]. Nitric oxide (NO) has several physiological functions as a biological messenger molecules. In addition, NO has a free radical metabolite that may cause oxidative stress and a variety of detrimental modifications in cells, including lipid peroxidation, protein functional abnormalities, DNA damage, and mitochondrial malfunction [17].

The *NOS1* gene has been linked to PD in several investigations done all over the world. PD risk variants in the *NOS1* gene have been examined by a research team from the Section of Medical Genomics, Department of Clinical Genetics, VU University Medical Center, Amsterdam, Netherlands. In their study, 209 PD patients and 488 controls of European (mostly French) origin were matched for age, sex, and place of domicile. They have noticed the involvement of exons 22 and 29 of the *NOS1* gene in the pathogenesis of PD. Overall, this research is in favor of the *NOS1* gene acting as a novel modifier gene in PD [18]. Researchers at SRM University in Kattankulathur, Tamil Nadu, India, have investigated the enhanced possibility of reliable molecular definition in PD identification during the early symptomatic phase of the illness. The microarray-based gene expression profiling of the *NOS1 *gene and other pathway-related genes was included in the study. This is the final window of opportunity for therapeutic action [19]. The risk of PD is inversely correlated with smoking, according to research from INSERM, U708 Neuroepidemiology, and University Pierre et Marie Curie, Paris, France, Groupe hospitalier Pitié Salpêtrière, Paris Cedex, France. Overall, the research found that the *NOS1 *gene contributes to PD susceptibility.

Although the precise mechanism behind the connection between smoking and hereditary variables is still unknown and calls for additional research, the evidence points to cigarette smoking having a protective effect against PD [20]. Researchers from Duke University claimed that the *NOS1* gene's SNPs rs3782218, rs11068447, rs7295972, rs2293052, rs12829185, rs1047735, rs3741475, and rs2682826 are linked to PD. They concluded that the *NOS1* gene is a significant genetic risk factor for PD [21].

Our investigation on PD patients from North Karnataka districts found two 3' UTR variants, rs1434015950 and rs2682826, which were recorded in earlier studies. Thus, in our study population, exon 29 of the *NOS1* gene is not responsible for PD. The present study targeted only a single exonic region, which was the limitation of our study. India is renowned for its diverse population, which includes many endogamous tribes that exhibit significant levels of inbreeding. This necessitates screening several families and a larger population to provide an accurate picture of the role of the *NOS1* gene and other linked genes in PD.

## Conclusions

The present study showed the absence of coding sequence mutations in the exon 29 of the *NOS1 *gene in the North Karnataka PD patients. Thus, an in-depth analysis of other exons of the *NOS1* gene or other genes involved in the pathogenesis of PD is needed using whole genome sequencing to understand the genetics of PD. These investigations will reveal the precise genetic causes of PD and its pathogenesis. Accordingly, more research is required to comprehend the genetic foundation of PD.

## References

[1] Parkinson J (2002). An essay on the shaking palsy. J Neuropsychiatry Clin Neurosci.

[2] Antoniou N, Prodromidou K, Kouroupi G (2022). High content screening and proteomic analysis identify a kinase inhibitor that rescues pathological phenotypes in a patient-derived model of Parkinson's disease. NPJ Parkinsons Dis.

[3] Engelhardt E, Gomes MD (2017). Lewy and his inclusion bodies: Discovery and rejection. Dement Neuropsychol.

[4] Mhyre TR, Boyd JT, Hamill RW, Maguire-Zeiss KA (2012). Parkinson's disease. Subcell Biochem.

[5] Kern DS, Kumar R (2007). Deep brain stimulation. Neurologist.

[6] Klein C, Westenberger A (2012). Genetics of Parkinson's disease. Cold Spring Harb Perspect Med.

[7] Schiesling C, Kieper N, Seidel K, Krüger R (2008). Review: Familial Parkinson's disease--genetics, clinical phenotype and neuropathology in relation to the common sporadic form of the disease. Neuropathol Appl Neurobiol.

[8] Helmich RC, Hallett M, Deuschl G, Toni I, Bloem BR (2012). Cerebral causes and consequences of parkinsonian resting tremor: a tale of two circuits?. Brain.

[9] Nuytemans K, Theuns J, Cruts M, Van Broeckhoven C (2010). Genetic etiology of Parkinson disease associated with mutations in the SNCA, PARK2, PINK1, PARK7, and LRRK2 genes: a mutation update. Hum Mutat.

[10] Moran LB, Croisier E, Duke DC (2007). Analysis of alpha-synuclein, dopamine and parkin pathways in neuropathologically confirmed parkinsonian nigra. Acta Neuropathol.

[11] Rachakonda V, Pan TH, LE WD (2004). Biomarkers of neurodegenerative disorders: how good are they?. Cell Res.

[12] Lesage S, Brice A (2009). Parkinson's disease: from monogenic forms to genetic susceptibility factors. Hum Mol Genet.

[13] Beyer K, Domingo-Sàbat M, Ariza A (2009). Molecular pathology of Lewy body diseases. Int J Mol Sci.

[14] Recchia A, Debetto P, Negro A, Guidolin D, Skaper SD, Giusti P (2004). Alpha-synuclein and Parkinson's disease. FASEB J.

[15] Kadakol GS, Kulkarni SS, Wali GM, Gai PB (2014). Molecular analysis of α-synuclein gene in Parkinson's disease in North Karnataka, India. Neurol India.

[16] Karimi M, Golchin N, Tabbal SD (2008). Subthalamic nucleus stimulation-induced regional blood flow responses correlate with improvement of motor signs in Parkinson disease. Brain.

[17] National Human Genome Research Institute (2012). American Society of Human Genetics' meeting showcases NHGRI research. https://www.genome.gov/26023267/american-society-of-human-genetics-meeting-showcases-nhgri-research.

[18] Levecque C, Elbaz A, Clavel J (2003). Association between Parkinson's disease and polymorphisms in the nNOS and iNOS genes in a community-based case-control study. Hum Mol Genet.

[19] Ahmed SS, Santosh W, Kumar S, Christlet HT (2009). Metabolic profiling of Parkinson's disease: evidence of biomarker from gene expression analysis and rapid neural network detection. J Biomed Sci.

[20] Elbaz A, Dufouil C, Alpérovitch A (2007). Interaction between genes and environment in neurodegenerative diseases. C R Biol.

[21] Hancock DB, Martin ER, Vance JM, Scott WK (2008). Nitric oxide synthase genes and their interactions with environmental factors in Parkinson's disease. Neurogenetics.

